# Potential Role of the Microbiome in Acne: A Comprehensive Review

**DOI:** 10.3390/jcm8070987

**Published:** 2019-07-07

**Authors:** Young Bok Lee, Eun Jung Byun, Hei Sung Kim

**Affiliations:** 1 Department of Dermatology, Uijeongbu St. Mary’s Hospital, The Catholic University of Korea, Seoul 06591, Korea; 2 Department of Dermatology, Incheon St. Mary’s Hospital, The Catholic University of Korea, Seoul 06591, Korea; 3 Department of Biomedicine & Health Sciences, The Catholic University of Korea, 222 Banpo-daero, Seocho-gu, Seoul 06591, Korea

**Keywords:** acne, microbiota, microbiome, skin, gut, brain, therapeutic implications

## Abstract

Acne is a highly prevalent inflammatory skin condition involving sebaceous sties. Although it clearly develops from an interplay of multiple factors, the exact cause of acne remains elusive. It is increasingly believed that the interaction between skin microbes and host immunity plays an important role in this disease, with perturbed microbial composition and activity found in acne patients. *Cutibacterium acnes* (*C. acnes*; formerly called *Propionibacterium acnes*) is commonly found in sebum-rich areas and its over-proliferation has long been thought to contribute to the disease. However, information provided by advanced metagenomic sequencing has indicated that the cutaneous microbiota in acne patients and acne-free individuals differ at the virulent-specific lineage level. Acne also has close connections with the gastrointestinal tract, and many argue that the gut microbiota could be involved in the pathogenic process of acne. The emotions of stress (e.g., depression and anxiety), for instance, have been hypothesized to aggravate acne by altering the gut microbiota and increasing intestinal permeability, potentially contributing to skin inflammation. Over the years, an expanding body of research has highlighted the presence of a gut–brain–skin axis that connects gut microbes, oral probiotics, and diet, currently an area of intense scrutiny, to acne severity. This review concentrates on the skin and gut microbes in acne, the role that the gut–brain–skin axis plays in the immunobiology of acne, and newly emerging microbiome-based therapies that can be applied to treat acne.

## 1. Introduction

The term ‘microbiome’ covers a whole range of micro-organisms, including bacteria, viruses, and fungi, their genes and metabolites, and the environment surrounding them. The word ‘microbiota’ is more confined, describing the group of commensals, symbiotic, and pathogenic micro-organisms found in a fixed environment. The number of microbial cells colonizing the human body is striking, being 10 times the number of human cells. Aside from the number, researchers are starting to appreciate that the indigenous microbes of the skin and gut are vital to the immunologic, hormonal, and metabolic equilibrium of the host [1,2].

Acne is an inflammatory condition involving the pilosebaceous unit that affects up to 90% of teenagers. Severe forms of acne can cause disfiguration and scarring, resulting in low self-esteem, difficulties in social interaction, and psychological distress. Increased sebum production, inflammatory mediators of the skin, and follicular keratinization of the pilosebaceous ducts are believed to contribute to acne development. Colonization by *Cutibacterium acnes* (*C. acnes*; formerly called *Propionibacterium acnes*) is also recognized in acne patients, but its role is unclear because it is ubiquitous in the sebaceous areas of healthy skin from puberty onward. As part of the rising interest in the human microbiome, study findings have begun to clarify how skin microorganisms participate in health and disease (i.e., acne).

Emerging data suggest that dietary factors (i.e., the Western diet) may influence acne development. A typical Western pattern diet which includes foods with complex mixture of fat (i.e., red meat), high glycemic index, and dairy may aggravate acne by raising the levels of insulin-like growth factor-1 (IGF-1) and insulin [3,4,5,6]. Diet also shapes the gut microbiota. A large body of evidence indicates that a low fiber-high fat Western diet causes fundamental changes in the intestinal microbiota, producing metabolic and inflammatory skin diseases [7].

In this review, we discuss host–microbe interactions in acne to clarify understanding of the disease and enable better treatments.

## 2. Methods

In September 2018, we searched the MEDLINE (1946 to present) database for publications covering the microbiome in acne. To search for relevant papers, the following keywords were used: “microbiome”, “microbiota”, “skin”, “gut”, “pathogenesis”, “*Cutibacterium* (*Propionibacterium*) *acnes*”, “oral antibiotics”, “isotretinoin”, “treatment”, “probiotics”, and “acne”. Inclusion criteria were original reports (human, animal, and cell studies) and review papers on microbiome in acne. Bibliographies were searched for additional studies that met the inclusion criteria. Studies in languages other than English, meeting abstracts, and posters were excluded.

## 3. Skin Microbiota (Overview)

Human skin, which covers an area of 2 m^2^ in adults, is the body’s largest organ and provides the first line of defense against external agents. The skin functions as both a physical and immunological barrier, performing a wide range of innate and adaptive immune functions [8]. Resident skin microbes stabilize the host’s barrier by fighting off pathogens, interacting with immune cells in the skin [9], and modifying host immunity [10]. Therefore, the skin microbiota is as an essential part of human health, and dysbiosis is thought to cause or aggravate skin diseases [11]. Advances in sequencing technology, such as 16S ribosomal RNA (16S rRNA) gene sequencing, have provided tremendous insights into the human microbiome.

### 3.1. Skin Microbiome Sampling

**Figure 1:**
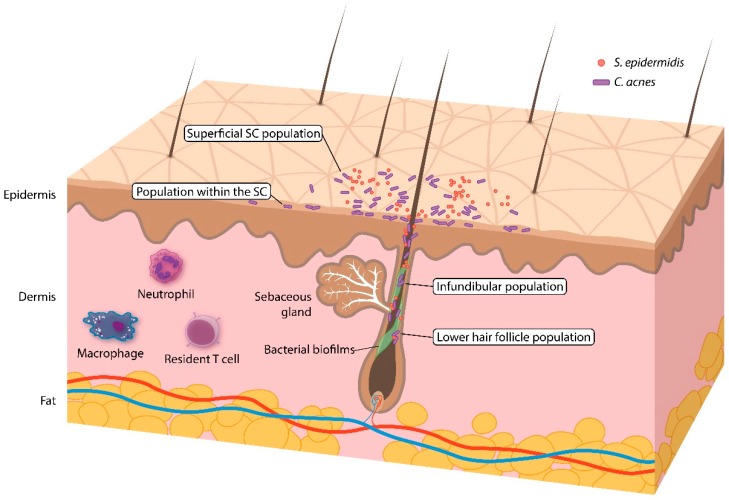
Overview of the skin (pilosebaceous unit) and the *C. acnes* population within it.

Acquiring an accurate and representative sample is a major challenge in studying the skin microbiome. Four sampling methods have been popularly documented in prior studies: skin swabbing, scraping, pore stripping, and punch biopsies (Table 1). Among the different methodologies, swabbing is the most practical, being simple, quick, and noninvasive. However, swabbing might not correctly reflect the microbiota across all skin layers. Skin scraping offers the benefits of collecting skin cells and their associated microbes. Pore stripping with a pliable, adhesive tape, or cyanoacrylate glue collects follicular contents, and can be useful in acne studies. A punch biopsy samples all three layers of the skin and might best represent the skin microbiota (Figure 1). Unfortunately, it is also intrusive and covers a smaller area than the other sampling methods. The diversity profile of human skin microbes has been compared among the different sampling methods. Grice et al. reported a 97% match in operational taxonomic units (phylotypes), regardless of the sampling method (swab, scrape, punch biopsy) [12]. Hall et al. [13] found that *C. acnes* was identified equally by swabbing, commercial pore strips, and a cyanoacrylate glue follicular biopsy, suggesting that the sampling method does not alter *C. acnes*-related characteristics and that all methods are appropriate for acne research. However, Prast-Nielsen et al. [14] recently demonstrated that skin swabs and skin biopsies produce different microbial profiles.

### 3.2. Skin Microbiome Analysis

**Figure 2:**
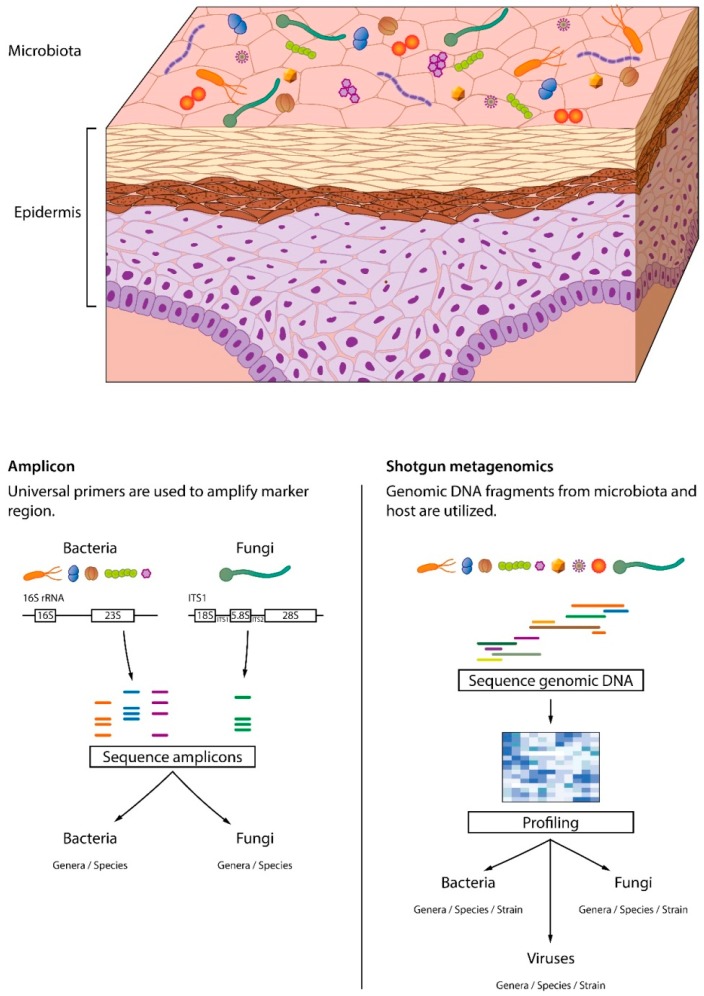
Amplicon and whole genome (shotgun) metagenomic sequencing. In amplicon sequencing, primers are used to amplify marker regions. Whole genome sequencing captures all the genetic information within a sample. Only shotgun metagenomics can identify viruses and offer resolution at the strain level.

Early studies of the skin microbiota used culture-based methods for bacterial identification and characterization. Microbial communities described by culture-based approaches are insufficient, with less than 1% of bacterial species being cultured. Furthermore, those studies have selectively focused on coagulase-negative staphylococci and *Propionibacterium* to reduce labor time, making their outcomes even more incomplete. To overcome those limitations, culture-independent microbial DNA-based approaches have been introduced (Figure 2). Among them, 16S rDNA amplicon sequencing has been a major breakthrough in bacterial identification, enabling bacterial differentiation at the species level. Although useful for taxonomic assignment, it is time-consuming. Another well-known culture-independent approach is shotgun metagenomics, which uses all the DNA present in the sample for sequencing [15]. Because shotgun sequencing analyzes the diverse gene content within a sample, multi-kingdom strain-level resolution is possible, and even the functional properties of communities are captured [16]. Despite those benefits, shotgun sequencing faces several challenges. First, it requires large amounts of DNA for analysis which is difficult to obtain using the typical noninvasive sampling techniques. Second, the metagenomic data are complex and large, which complicates the informatic analysis. Additional challenges are the need to implement a database with reference genomes and high cost [17]. Nonetheless, shotgun metagenomics (untargeted) sequencing provides more in-depth information than amplicon-based profiling approaches.

### 3.3. Human Skin Microbiota (Healthy Skin)

As the major inhabitant of the skin, bacteria are the best studied parts of the skin microbiota. Most commensal skin bacteria are categorized into the following four phyla: Actinobacteria (i.e., Corynebacterineae, Propionibacterineae), Proteobacteria, Firmicutes (i.e., Staphylococcaceae), and Bacteroidetes [18]. The bacterial composition differs from person to person and varies according to the body site [19,20,21]. Environmental factors such as the use of soaps, cosmetics, antibiotics, occupation, temperature, humidity, and UV exposure [22] also influence microbial colonization [23,24].

**Figure 3:**
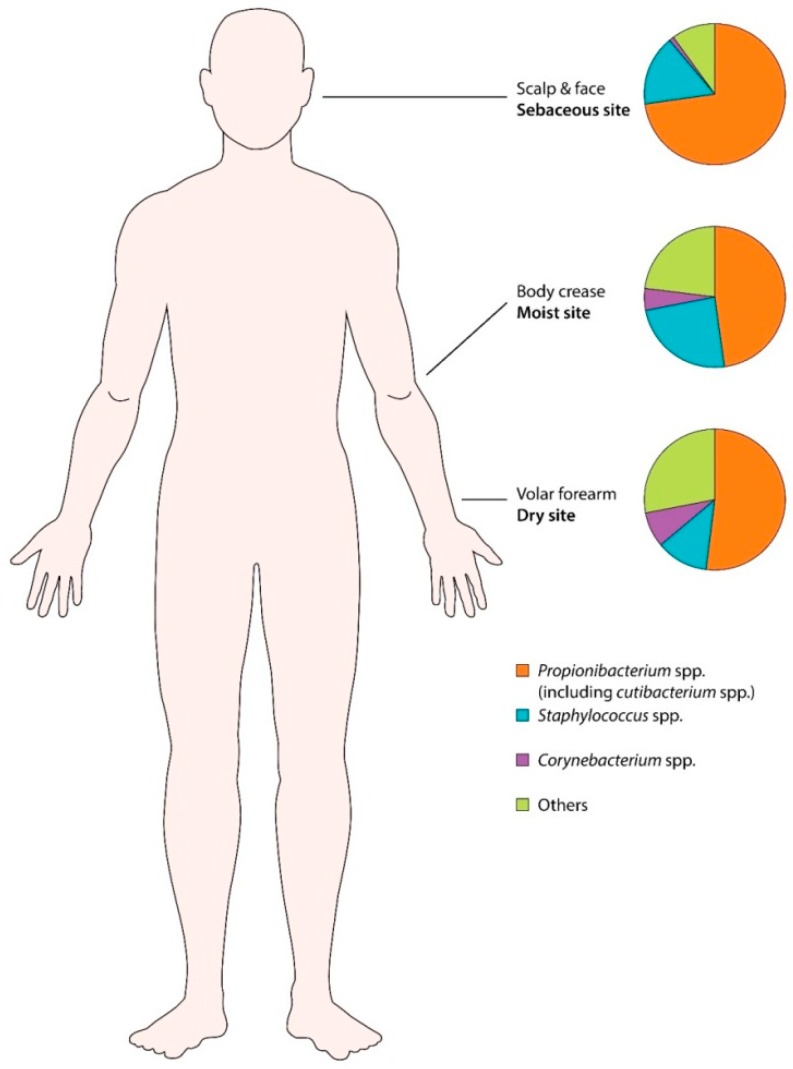
Human skin microbiota in different body sites (moist, sebaceous, and dry). *Propionibacterium* spp. (including *Cutibacterium* spp.) are most prevalent in sebum rich areas.

Body sites are divided into three categories: moist, sebaceous, and dry (Figure 3). The microbes preferentially found in moist areas (i.e., axillae, inguinal area, sole, popliteal fossa) are *Staphylococcus* and *Corynebacterium* species. Sebaceous sites such as the forehead, retro-auricular area, back, and alar crease, show the lowest bacterial diversity, indicating that only a small subset of organisms can tolerate this condition. *Propionibacterium* (including *Cutibacterium*) species are the main isolates from sebaceous areas, because they can survive in an anaerobic, lipid-rich condition. Dry areas of the skin (i.e., forearm) have the most diverse microbial community, carrying a mixture of the four major phyla [25].

Skin microbial composition changes with age. In neonates, the microbiota on the skin largely depends on the route of delivery (vaginal vs. cesarean section), and in infancy, Firmicutes becomes dominant [26]. The microbiota of the sebaceous areas (i.e., face) takes shape during puberty as hormonal changes activate the sebaceous glands [19]. Notably, interpersonal variation is greater than the changes within the same person over time [27]. Gender is also an important host factor that influences bacterial composition and diversity. Women’s hand surfaces and forearms are colonized by a more diverse set of microbes than men’s, whereas men carry more *Malassezia* than women [23]. Such a gender difference could be partially explained by behavioral habits, such as the use of make-up [28].

## 4. Skin Microbiota and Acne

Since its first observation in acne lesions by Unna (1896) and its isolation by Sabouraud (1897), *C. acnes* has been considered the likeliest pathogen of acne. *C. acnes* was originally named *Bacillus acnes*, and subsequently renamed *Corynebacterium acnes* because it is morphologically akin to *Corynebacterium*. It was labeled *P. acnes* in the 1940s because of its ability to produce propionic acid. In 2016, a novel genus, *Cutibacterium* was proposed for a *Propionibacterium* subset (cutaneous *Propionibacterium*), and thus *P. acnes* is now called *Cutibacterium acnes (C. acnes)* [29].

*C. acnes* is the major occupant of the pilosebaceous unit, accounting for up to 90% of the microbiota in sebum rich sites such as the scalp, face, chest, and back [30]. The scalp and face carry the highest density of *C. acnes,* followed by the upper limbs and trunk, with lower limbs showing the least *C. acnes* [30,31]. The abundance of *C. acnes* also changes with age. *C. acnes* is scarce on the skin in childhood, gradually increases from puberty to adulthood and then decreases after the age of 50 years.

Although the role of *C. acnes* in the pathophysiology of acne remains uncertain, *C. acnes* is primarily known as a beneficial commensal. It helps maintain a low skin pH by releasing free fatty acids, and it blocks pathogens (i.e., *Staphylococcus aureus* and *Streptococcus*) from colonizing the skin [32].

### 4.1. Classification of C. acnes

*C. acnes* is a major skin commensal in both acne patients and acne-free individuals. It is worth mentioning that excess *C. acnes* colonization might not be an important factor in acne pathogenesis with some studies reporting little difference in the comparative amount of *C. acnes* in individuals with and without acne [33]. Recent studies suggest that *C. acnes* acts as a pathogen or a commensal according to the strain and balance among the metagenomic elements [33,34].

Initially, *C. acnes* was grouped into two types, I and II, based on their cell wall sugar content, serum lectin response, and vulnerability to phages [35]. Later, a new phylotype, type III, with long slender filaments was found [36]. Now some researchers have proposed reclassifying phylotype I as *C. acnes* subsp. *acnes* [37], phylotype II as *C. acnes* subsp. *defendens* [38,39], and phylotype III as *C. acnes* subsp. *elongatum* [40].

Because *C. acnes* has a striking clonal population structure with relative sequence preservation, multi-locus sequence typing (MLST) is needed for higher strain typing resolution. Within type I, *C. acnes* is further divided into clades IA1, IA2, IB, and IC according to the Belfast MLST program [41] and I-1a, I-1b, and I-2 in the Aarhus MLST measure [42]. Whole genome sequencing has been able to provide a higher-resolution phylogeny of *C. acnes* [43]. Using its gene-based single nucleotide polymorphisms, *C. acnes* is stratified into phylogenetic clades IA-1, IA-2, IB-1, IB-2, IB-3, IC, II, and III [33,43]. Table 2 presents the names of the phylogenetic clades (*C. acnes* strains) from whole-genome sequencing and different MLST programs. An alternative process was described by Fitz-Gibbon et al. [33] who performed 16S rRNA gene sequencing as well as complete genome sequencing to classify *C. acnes* into ribotypes (RTs). These classifications can distinguish the presence of healthy and acne-related *C. acnes* strains. Strains from clades IA-2 (mostly RT4 and RT5), IB-1 (RT8) and IC (RT5) are closely linked with acne. Type II strains that include RT2 and RT6 are often found in healthy (acne-free) skin. Strains from clades IA-1, IB-2, and IB-3 are associated with both acne-free individuals and acne patients [33,43,44] (Table 2). Type III strains are rare on the face but profuse on the trunk and connected to progressive macular hypomelanosis [29,45,46].

Recent metagenomic studies have cast new light on the strain-level differences of *C. acnes* in health and disease (acne). Tomida et al. [43] compared the full DNA sequences of *C. acnes* strains to find that the non-core genomic sector of acne-related *C. acnes* strains carry extra virulence genes compared with healthy strains. Johnson et al. [47] identified that acne-related strains generate more porphyrin, a substance that generates reactive oxygen species (ROS) and can stir up inflammation in keratinocytes [48]. It was further demonstrated that *C. acnes* strains respond differently to vitamin B12 supplements—porphyrin spikes in acne-related strains, whereas the level remains largely the same in healthy strains [47,49]. Acne-associated *C. acnes* strains are also known to induce an inflammatory response in sebocytes, keratinocytes, and peripheral blood mononuclear cells, whereas healthy strains do not [50,51,52,53].

### 4.2. Cutibacterium acnes in Acne

**Figure 4:**
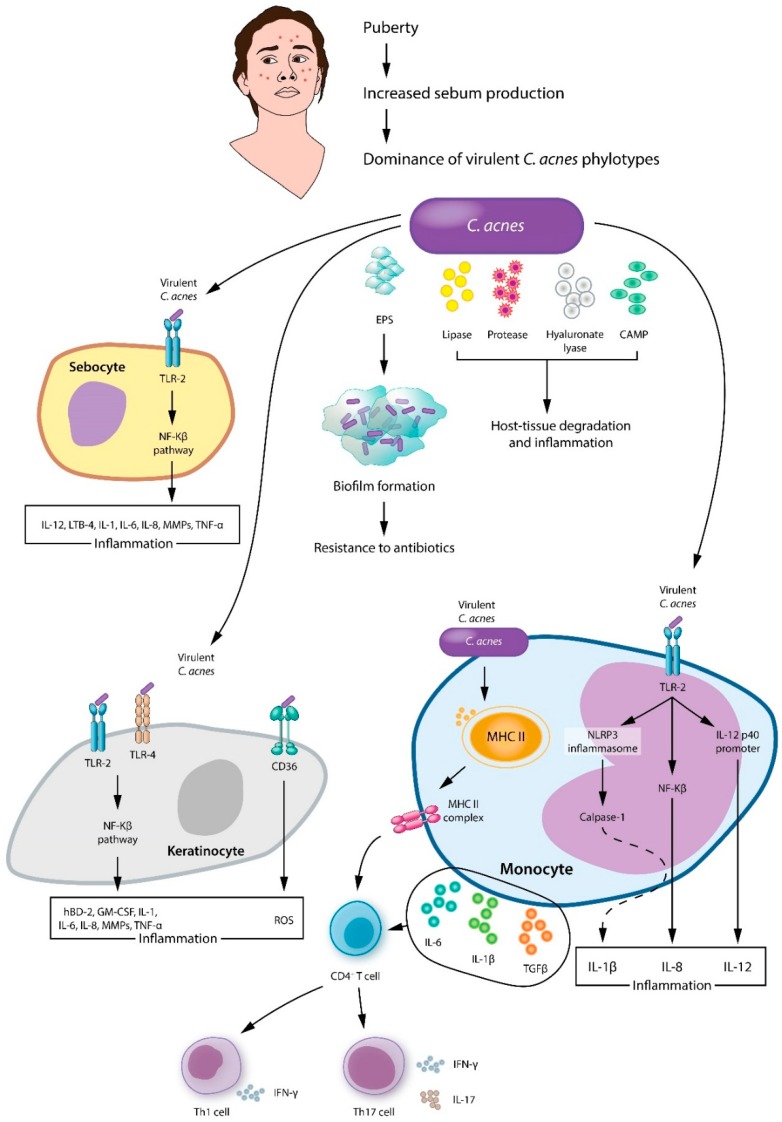
A proposed model of main pathologic processes induced by *C. acnes* involve sebocytes, keratinocytes, and monocytes in acne vulgaris. EPS: extracellular polymeric substances; CAMP: cyclic adenosine monophosphate; TLR: toll like receptor; IL: interleukin; TNF: tumor necrosis factor; LTB: leukotriene B; CD36: cluster of differentiation 36; GM-CSF: granulocyte-macrophage colony stimulating factor; hBD: human β-defensin; MMPs: matrix metalloproteinases.

*C. acnes* is an aerotolerant, anaerobic, Gram-positive, pleomorphic rod that belongs to the Actinobacteria phylum. Several mechanisms have been proposed by which *C. acnes* aggravates acne, including augmentation of lipogenesis, comedone formation, and host inflammation [29] (Figure 4). As for lipogenesis, *C. acnes* encouraged hamster sebocytes to synthesize lipid droplets and triacylglycerol. In addition, its application to hamster auricles induced sebum to accumulate [54]. *C. acnes* promotes comedogenesis by generating oxidized squalene and free fatty acids, causing a qualitative change in sebum [55,56]. In addition, *C. acnes* activates the IGF-1/IGF-1 receptor signaling pathway to upregulate filaggrin expression, which increases integrin-α3, -α6, and vβ6 levels, thereby affecting keratinocyte proliferation and differentiation and resulting in comedone formation [57,58]. Last but not least, *C. acnes* induces and aggravates inflammation. *C. acnes* activates Toll-like receptors (TLR-2 and TLR-4) on keratinocytes, which leads to the activation of the MAPK and NF-kB pathways. Subsequently, keratinocytes produce interleukin (IL)-1, IL-6, IL-8, tumor necrosis factor-α, granulocyte-macrophage colony stimulating factor, human β-defensin-2, and matrix metalloproteinases [50,59,60]. Next to TLR-2 and TLR-4, CD-36 recognizes *C. acnes* [61] and stimulates ROS production from keratinocytes to wipe out bacteria and induce inflammation [62]. Sebocytes are also involved in the inflammatory response. As in keratinocytes, sebocyte TLR-2 recognizes *C. acnes* and activates the NF-kB pathway, resulting in inflammation [63]. *C. acnes* is also spotted by the TLR-2 expressed on monocyte/macrophage lineage cells, which results in the production of the inflammatory cytokines IL-8 and IL-12 [6]. In addition, *C. acnes* stimulates the gene expression of caspase-1 and NLRP3 inflammasome in monocytes, causing an excess of IL-1β [64]. *C. acnes* also has T cell mitogenic activity [65]. The *C. acnes*-induced adaptive immune response involves CD4+ T lymphocytes, specifically T helper (Th)1 and Th17 cells [66]. *C. acnes* provokes peripheral blood mononuclear cells to secrete IL-6, IL-1β, and transforming growth factor-β and encourages naive CD4+CD45RA T lymphocytes to differentiate into Th1 and Th17 cells [66,67]. As a result, Th effector cytokines such as IL-17 and interferon-γ are upregulated [68].

*C. acnes* strains that are highly virulent and resistant to antibiotics (i.e., RT4, 5, 10), are dominant on acne patients’ skin [33,69]. Virulence factors such as lipase, protease, hyaluronate lyase, endoglycoceramidase, neuraminidase, and Christie–Atkins–Munch-Petersen (CAMP) factor cause host-tissue degradation and inflammation. Lipase chemoattracts neutrophils and hydrolyzes sebum triglycerides to free fatty acids, inducing inflammation and hyperkeratosis [70,71,72]. Protease, hyaluronate lyase, endoglycoceramidase, and neuraminidase have degrading properties and aid the invasion of *C. acnes* by breaking down extracellular matrix constituents [73,74,75]. As the extracellular matrix resolves, inflammatory cells (i.e., neutrophils, monocytes, dendritic cells,) invade the hair follicle, bringing about the effusion of bacteria, keratin, and sebum to the dermis, which provokes foreign body granuloma and scarring [76].

Another pathological feature of *C. acnes* is biofilm [60], an aggregate of microbiota embedded within a self-made matrix of extracellular polymeric substance (EPS), adhered to a surface. The structural properties and physiology of biofilm bacteria confer resistance to antibacterial agents and host inflammatory cells. Whole genome sequencing of *C. acnes* has provided evidence of the formation of biofilms. Type I strains associated with acne carry a novel linear plasmid with a tight adhesion locus that is essential for biofilm formation, colonization, and virulence [33,77]. Skin biopsies have provided further evidence, with a greater degree of follicular *C. acnes* colonization and biofilm found in acne samples compared with controls [78]. As for intrinsic antimicrobial resistance, EPS acts as a diffusion barrier and delays the inflow of antimicrobial agents, by chemically antagonizing the antimicrobial molecules or limiting antimicrobial transport. Another mechanism in biofilm resistance is the reduced growth rate and metabolism of biofilm-associated organisms, which slows their take-up of antimicrobial agents [79]. In terms of acquired resistance, transfer of resistance-conferring plasmids takes place by conjugation among the different organisms within a biofilm [80].

### 4.3. Other Acne-Associated Microbiota

Human skin is colonized by a wide variety of microbes, which function in maintaining skin health or exacerbating disease. Although *C. acnes* is best-known for its connection with acne, it is speculated that other bacteria might also (indirectly) contribute to the inflammatory process (Table 3). Rajiv et al. [81] observed that *C. acnes* and *S. epidermidis* were more prevalent in acne patients, than in the control population. The applicability of the finding was tested using explant models and *S. epidermidis* was found to prevent acne and exert antimicrobial activity. Recently, it has been noted that *S. epidermidis* not only infects humans but also protects us from several pathogenic bacteria [82,83]. *S. epidermidis* has been reported to produce antimicrobial peptides such as epidermin, phenol-soluble modulins, Pep5, and epilancin (K7, 15X) [84,85,86]. Some studies hint that *S. epidermidis* can inhibit the growth of *C. acnes*. Wang et al. [87] claimed that *S. epidermidis* strains release succinic acid, which has an anti-*C. acnes* effect. Christensen et al. [88] reported that *S. epidermidis* secretes polymorphic toxins that inhibit *C. acnes* growth. In addition, *S. epidermidis* was shown to generate *staphylococcal* lipoteichoic acid, which dampens *C. acnes*-related inflammation by increasing the expression of miR-143 and blocking TLR-2 expression in keratinocytes [89]. Thus, *S. epidermidis* might play a role in acne prevention, but that hypothesis needs further testing.

Culture-based studies have reported that *C. granulosum* is highly abundant in the comedones and pustules of acne patients [90]. Furthermore, *C. granulosum* displays stronger virulence (i.e., lipase activity) than *C. acnes* [91]. On the other hand, the currently available genome data for this bacterium suggests a limited range of virulence-associated genes, with a striking absence of the CAMP factor, sialidase, and hyaluronate lyase that contribute to *C. acnes*–host interaction in acne. Further studies are needed to find the exact role of this minor *Cutibacterium* species in acne and health [92,93].

*Malassezia* is the most copious cutaneous fungal organism and has long been thought to cause acne [94]. According to a study by Hu et al. [95] treatment-resistant acne papules disappeared after the use of anti-fungal agents. Therefore, those authors argued that *Malassezia,* rather than *C. acnes* is associated with refractory acne. Several other study findings are in line with that theory. Song et al. [96] and Numata et al. [97] found that *Malassezia restricata* and *Malassezia globosa* are easily collected from young acne patients. Akaza et al. [98] revealed that the activity of *Malassezia* lipase is 100 times greater than that of *C. acnes. Malassezia* hydrolyzes sebum triglycerides into free fatty acids, which causes hyper-keratinization of hair follicular ducts and comedone formation [99]. It also chemo-attracts neutrophils and promotes the release of pro-inflammatory cytokines from monocytes and keratinocytes [100,101]. The involvement of *Malassezia* in the pathophysiology of acne remains to be clarified [29].

## 5. Acne Treatment and Skin Microbes

Because acne is a multifactorial inflammatory skin condition, a handful of treatment options are available, including topical and oral antibiotics, retinoids, and photodynamic therapy [102]. Acne is not a typical skin infection, but antibiotics have played a central part in acne treatment for more than 40 years. Topical antibiotics suppress *C. acnes* and act as an anti-inflammatory agent [103]. Oral antibiotics are best for moderate-to-severe acne, especially for those who fail to respond to or tolerate topical agents. According to the therapeutic guidelines and expert opinion, macrolides, clindamycin, and tetracyclines are the antibiotics of choice for acne [104,105,106,107]. Erythromycin, roxithromycin, clarithromycin, and azithromycin are macrolides. Clindamycin is a lincosamide antibiotic. Tetracyclines frequently used on acne are doxycycline, tetracycline, and minocycline.

Other popular agents that directly inhibit *C. acnes* colonization include benzoyl peroxide and azelaic acid [63]. The therapeutic effect of benzoyl peroxide comes from its mild comedolytic action and ability to kill *C. acnes* via the release of free oxygen radicals [108]. Topical azelaic acid has anti-inflammatory and antibacterial properties, in addition to its comedolytic effects [109].

Isotretinoin is an all-trans retinoic acid pro-drug and has been the final option for patients with severe recalcitrant acne [110]. Its repression of sebum production is well known, and it was recently found to normalize the *C. acnes*/TLR-2-mediated innate immune response in acne patients [111]. It is reasonable to think that isotretinoin indirectly affects skin microbes, because it blocks the supply of an essential nutrient and stabilizes the overzealous immune system, but few have examined this in detail. Both oral [112,113,114] and topical [115] retinoids were reported to bring down the number of *C. acnes* and cause changes in microbial diversity in patients with acne. In a recent study by McCoy et al., isotretinoin was shown to have varying effects on the *Propionibacterium* subtaxa [116].

Alternative acne treatments include blue light, ultraviolet (UV) phototherapy, and photodynamic therapy [117,118,119]. These approaches could improve acne by reshaping the skin microbiota and lowering *C. acnes* counts within the lesions. UV light is a well-documented bactericidal treatment [22] that can block the release of lipoteichoic acid, lipopolysaccharides, and other bacterial metabolites with pro-inflammatory effects.

### 5.1. Antibiotics and the Acne Skin Microbiota

Few studies have examined the effects of antibiotics on the skin microbiota in acne. According to culture-based research, tetracycline antibiotics cause a decrease in the abundance of *Cutibacterium* in acne patients’ skin [120].

Recently, Chien et al. [121] examined the changes in the skin microbial composition of acne patients following oral antibiotic therapy (sampling method: swabbing). Using 16S rRNA gene sequencing, *C. acnes* was found to be dominant at baseline. Four weeks of minocycline caused a 1.4-fold reduction of *C. acnes* in acne patients, with a recovery to baseline *C. acnes* levels 8 weeks after stopping the antibiotic. In addition to *C. acnes* reduction, there was a 5.6-fold increase in *Pseudomonas* species after taking the antibiotics for 4 weeks. The growth of *Pseudomonas* explains the opportunistic skin infections (i.e., gram-negative folliculitis) common among acne patients receiving prolonged antibiotic therapy. A 4.7-fold decrease in *Lactobacillus* relative to baseline was found 8 weeks following minocycline cessation, but whether that presents an increased risk for skin infections needs to be determined.

A 16S rRNA sequencing study by Kelhala et al. [112], reported that lymecycline treatment decreases the abundance of *Cutibacterium* in skin with acne (sampling method: swabbing). They also claimed that the skin microbiota became more diverse after lymecycline treatment. Alpha diversity is supposedly increased as a result of the moderation of *Cutibacterium* colonization, which allows other flora to become more visible.

### 5.2. Antibiotic Resistance in the Microbiota of Skin with Acne

Topical and oral antibiotics have been the center of acne treatment for a long time. *C. acnes* resistance to antibiotics has increased over the years and become a worldwide problem in acne patients, with higher rates of resistance being reported for clindamycin (lincosamide) (36–90%) and erythromycin (macrolide) (21–98%) than for tetracyclines (4–16%) [122,123,124,125,126]. This is in line with the fact that topical macrolides and clindamycin are the most commonly used. The molecular mechanisms that underlie erythromycin and clindamycin resistance are point mutations in the 23S rRNA, and the presence of an *erm*(X) gene, respectively [69,127,128,129,130]. Tetracycline resistance is associated with chromosomal point mutation in the 16s rRNA gene of *C. acnes* [69]. *C. acnes* becomes less susceptible to doxycycline when amino acid is substituted in the ribosomal S10 protein [131].

The degree of antibiotic resistance varies among the different *C. acnes* strains. It is well known that acne-associated phylotype IA1 strains (ribotypes R4, R5) are most often antibiotic-resistant [41,128,132]. Furthermore, McDowell et al. [41] reported that all the phylotype IC isolates (ribotype R5 strain) used in their study were resistant to erythromycin and tetracycline.

The use of clindamycin and macrolides in acne not only causes *C. acnes* to be resistant to antibiotics but also leads to increased drug resistance among other skin bacteria. Potential mechanisms include acquiring mobile genetic elements with multi-drug resistance genes through horizontal gene transfer between bacterial species. At least 30% of *S. epidermidis* isolated from skin with acne were resistant to roxithromycin, erythromycin, and clindamycin [133]. Little is known about the effect of tetracyclines on skin microbiota other than *C. acnes*.

With the regular use of antibiotics in acne, recommendations are made to minimize the risk of antimicrobial resistance [106,134]. First, antibiotics are not meant for comedones. As a baseline rule, the use of topical antibiotics alone must be avoided. Topical antibiotics, if necessary, should be combined with retinoid or benzoyl peroxide to lessen the risk of antimicrobial resistance [135]. For maintenance, topical retinoid is a good choice and benzoyl peroxide can be added for further defense against *C. acnes*. Azelaic acid is also great because it blocks cellular protein synthesis in *C. acnes* without causing bacterial resistance [106].

Systemic antibiotics, combined with a topical agent (i.e., benzoyl peroxide, tretinoin, azelaic acid) are preferable for moderate to severe inflammatory acne, but they should not be used for more than 3 months. Oral tetracyclines (i.e., lymecycline, doxycycline) are the antibiotics of choice for acne with high macrolide-resistance [106,134,136].

### 5.3. Skin Microbiota As a Biomarker for Acne Drug Development

Several studies have compared the skin microbiota of acne patients and acne-free individuals to find differences in the dominant *C. acnes* strains (more virulent stains were associated with acne) [33,34]. Acne treatment is also known to reduce the number of *C. acnes* on the skin and cause an increase in the diversity of skin bacteria [112,121]. Given the clear link between acne and *C. acnes*, the skin microbiota could be used as a biomarker for acne drug development and clinical trials [137].

## 6. Gut Microbiota and the Skin

The skin and gut, both heavily vascularized and richly innervated organs with critical neuroendocrine and immune functions, are somewhat similar [134]. Interestingly, mounting evidence suggests that the two organs have a bidirectional connection, and many studies link intestinal health to skin homeostasis and allostasis [92,138].

The gut contains an extensive collection of bacteria, fungi, viruses, and protozoa that outnumbers its host cells by 10-fold [139,140]. Recent advances in metagenomics have broadened our understanding on the intestinal microbiota and its influence in human health and disease [141]. The intestinal microbiota performs metabolic and immune functions, playing an essential role in the maintenance of physiological homeostasis. Gut flora break down food and indigestible complex polysaccharides, synthesize essential vitamins (vitamin K and biotin), and thus provide nutritional benefits to the host [142]. The gut microbiota also intricately regulates the host immune system, making possible both tolerance to dietary and environmental antigens and defense against potential pathogens [143].

Currently, strong evidence indicates that gut microbes play a mediating role between skin inflammation and emotion [7]. In 1930, Stokes and Pillsbury issued a ‘theoretical and practical consideration of a gastrointestinal mechanism’ by which the skin is altered by emotional and nervous states. Those authors linked emotions such as worry, anxiety, and depression to changes in gut microorganisms, which they proposed would promote focal and systemic inflammation (the brain–gut–skin theory) [144].

Although not yet fully known, the mechanism by which the gut microbiota influences skin homeostasis seems to come from its modulatory effect on systemic immunity [92]. In addition, evidence suggests that the gut flora can affect the skin more directly, by transporting the gut microbiota to the skin [92,145]. When the intestinal barrier is disrupted, gut microbiota and their metabolites quickly enter the bloodstream, accumulate in the skin, and disturb the skin equilibrium [92]. The gut microbiota may also affect the skin microbiota by generating short chain fatty acids (SCFAs), during fiber fermentation in the gut [143]. SCFAs such as propionic acid were shown to have a profound antimicrobial effect against USA 300, the most prevailing community-acquired methicillin-resistant *Staphylococcus aureus* and is suggested to play a role in shaping the skin microbiota, which may influence cutaneous immunity [146]. *S. epidermidis* and *C. acnes* are examples of skin commensals that endure wider SCFA shifts than other skin microorganisms.

### 6.1. Gut Microbiota and Acne

The intestinal flora is thought to influence acne, possibly by interacting with the mTOR pathway [147,148,149]. Metabolites from the gut microbiota may constitutively control cell expansion, fat metabolism, and other metabolic functions through the mTOR pathway [150]. The mTOR pathway itself may also affect the gut microbiota by controlling the intestinal barrier [151]. In cases of gut dysbiosis and a disturbed intestinal barrier, a positive feedback loop can be formed, which may amplify the host metabolism and inflammation [152,153,154,155]. Considering the possible role of mTORC1 in acne pathophysiology [156], the interaction between mTOR and gut microbiota may serve as a mechanism by which the intestinal flora aggravates acne.

The connection between acne and gastrointestinal dysfunction can originate in the brain. Supporting this hypothesis is the stress-induced aggravation of acne. Experimental animal and human studies have shown that stress impairs the normal gut microflora, most notably *Lactobacillus* and *Bifidobacterium* species [144]. Psychological stressors cause intestinal microbes to produce neurotransmitters (i.e., acetylcholine, serotonin, norepinephrine) that cross the intestinal mucosa to enter the blood stream, resulting in systemic inflammation.

In recent years, the role of environmental factors, especially the Western diet, has been raised in acne pathogenesis. The Western diet includes dairy products, refined carbohydrates, chocolate, and saturated fat, which may aggravate acne by activating nutrient-derived metabolic signals [157,158]. Evidence also indicates that the intestinal flora associated with the Western diet contribute to inflammatory skin diseases. For instance, high-fat diets reduce the level of gut flora and increase the concentration of lipopolysaccharides, causing systemic inflammation by impairing colonic epithelial integrity and barrier function, decreasing mucus layer thickness, and increasing the secretion of pro-inflammatory cytokines [159,160].

**Figure 5:**
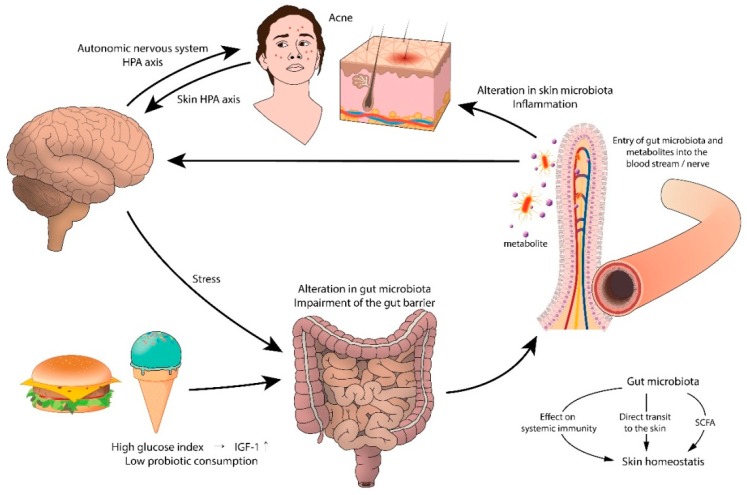
A proposed model of the gut–brain–skin axis in acne. HPA: hypothalamic pituitary adrenal; IGF-1: Insulin-like growth factor-1; SCFA: short chain fatty acid.

In 1930, Stokes and Pillsbury reported that a high proportion of acne patients had hypochlorhydria. Low acidity levels allow the relocation of colonic bacteria to the distal part of the small intestine, creating a state of gut dysbiosis and small intestine bacterial overgrowth [161,162], which causes increased intestinal permeability, and leads to skin inflammation [142] (Figure 5).

### 6.2. Gut Microbiota in Acne

Only a few researchers have examined the gut flora of acne patients. The first such study was conducted in 1955 and compared the presence of potentially pathogenic bacteria in 10 acne patients with that in acne-free individuals [163]. It is noteworthy that the *Bacteroides* species, which increase under stress conditions, were frequently isolated from acne patients. A Russian study reported that people with acne exhibit markedly different intestinal flora compared with acne-free controls [164].

In a study by Deng et al. [159], acne patients exhibited lower gut microbiota diversity and a higher ratio of *Bacteroidetes* to *Firmicutes,* which is an enterotype of the Western diet. In addition, Yan et al. [165] found a decrease in *Lactobacillus, Bifidobacterium, Butyricicoccus, Coprobacillus,* and *Allobaculum* in acne patients compared with controls, which provides a new understanding of the link between acne and the alteration of gut flora. *Lactobacillus* and *Bifidobacterium* are common probiotic species that balance the intestinal microbiota by fermenting unabsorbed oligosaccharides in the upper gut [166]. They also strengthen the intestinal barrier by decreasing permeability and enhancing the epithelial resistance of the gut [167]. In addition, *Bifidobacterium* and *Lactobacillus* encourage the production of CD4+Foxp3+T cells (regulatory T cells), and regulatory dendritic cells, suppressing T helper cell and B cell response and cytokine production [168]. *Butyricicoccus* generates butyrate, which provides energy to cells and prevents mucosal barrier damage and inflammation [169].

Further studies should be performed to identify the enteral flora of acne patients and find changes in the gut microbiota following acne therapy (i.e., oral antibiotics and isotretinoin). In a murine study [170], doxycycline caused long term changes in the intestinal microorganism. Isotretinoin did not have a significant effect on fecal microbes. On the other hand, isotretinoin did not have a significant effect on the fecal microbes in mice [170].

## 7. Probiotics and the Skin

Probiotics are living microorganisms that are beneficial to the host’s health. Upon ingestion, they provide a protective shield across the intestinal mucosa [171]. The most commonly used and therefore, the best studied probiotic strains to date are *Lactobacillus* and *Bifidobacterium*. The official definition of prebiotics is a non-digestible food component that benefits the host by stimulating the growth or activity of bacterial species present in the colon [172]. Although oral probiotics/prebiotics have been used in the past to prevent and treat bowel disease, evidence suggests that by adjusting the composition of the microbial community, probiotics induce immune reactions that expand beyond the gut to act on the skin [173,174]. Oral probiotics have been reported to enhance insulin sensitivity in animal models [175] and regulate skin inflammation by interacting with gut-associated lymphoid tissue [176]. Certain strains of *Lactobacillus* encourage the production of IL-10 (an anti-inflammatory cytokine) and promote T-regulatory cell function, which suggests that probiotics help balance the immune system in response to stimuli [176]. *Bifidobacterium coagulans (B. coagulans)* also has immune-regulatory properties that can affect skin health. Incubating polymorphonuclear cells and peripheral blood mononuclear cells with *B. coagulans* supernatant and cell wall fragments promoted the maturation of antigen presenting cells and blocked ROS formation [177,178].

### Probiotics and Acne

Growing evidence indicates that probiotics modify the pathophysiologic factors that contribute to acne, potentially improving patient compliance [174]. Probiotics directly inhibit *C. acnes* with antimicrobial proteins. In an in vitro study, *Streptococcus salivarius* suppressed the growth of *C. acnes* by secreting a bacteriocin-like inhibitory substance [179]. Similarly, strains of *Lactococcus sp. HY449* blocked *C. acnes* through the release of bacteriocins [180]. Probiotics, when topically applied, also improved the skin barrier and produced a secondary increase in antimicrobial peptides. *Streptococcus thermophiles* for instance, was shown to enhance ceramide production both in vitro and in vivo when applied as a cream for a week [181,182,183]. Ceramides are well-known for trapping water in the skin, but other than that, certain ceramide sphingolipids (i.e., phytosphingosine) display antimicrobial activity against *C. acnes,* thereby improving acne [184]. By producing ceramides, probiotics help strengthen the skin barrier, which is beneficial to acne patients because it calms the irritation caused by topical agents.

Probiotics also have immunomodulatory properties on keratinocytes and epithelial cells. *S. salivarius* strain K12 inhibited the release of pro-inflammatory cytokine IL-8 from keratinocytes [185]. Likewise, *L. paracasei* NCC2461 suppressed substance P-induced skin inflammation in human skin cultures [186,187]. Because substance P is involved in sebum production and acne inflammation, its suppression by probiotics suggests that probiotics could work as an adjunct in acne treatment [174,188]. IGF-1 is thought to participate in acne development. Foods rich in dairy and carbohydrates increase the risk of acne, probably by elevating IGF-1 [3,189]. Adding *Lactobacillus* to fermented milk caused a 4-fold decrease in IGF-1 compared with nonfermented skim milk [190]. Thus, probiotics could improve acne by regulating the IGF-1 level.

Clinical trials have assessed the effect of probiotics on acne. Kang et al. [191] reported that 8 weeks of topical *Enterococcus faecalis* treatment resulted in a 50% reduction in inflammatory acne count compared with placebo. A 5% extract of *Lactobacillus plantarum* also reduced acne severity (i.e., acne size, count, and associated erythema) [192]. In an Italian study [193], the group that received oral probiotics (250 mg of freeze-dried *Bifidobacterium bifidum* and *L. acidophilus*) as a supplement to acne treatment showed greater resolution of acne compared with the non-supplemented group. In addition, patients with probiotics supplementation showed greater tolerance of and compliance with oral antibiotics. A recent clinical trial [194] also indicated that probiotics decrease the side effects (i.e., vaginal candidiasis) associated with systemic antibiotics (i.e., minocycline) while providing synergistic benefits for inflammatory acne. Taken together, the findings suggest that the microbiota plays an important role in acne pathogenesis and can be modulated for clinical improvement, but efforts should be made to identify the exact mechanisms and therapeutic effects of oral/topical probiotics in acne (Table 4).

## 8. Conclusions

Using advances in technology, researchers have increased what is known about the human microbiome. Each person’s microbial environment is complex and individualized. Researchers have sought the link between microbiota and *C. acnes* for the past 100 years. Recent metagenomic studies have shown that acne vulgaris is characterized by the dominance of virulent strains of *C. acnes,* but the limitations and biases of current skin sampling methods indicate the need for a better approach. Also, given the growing number of patients who are treatment resistant, longitudinal assessments are needed on phenotypic changes in the skin microbiome with isotretinoin and antibiotic treatment. Until recently, diet and psychological stress were thought to have little relevance to the pathophysiology of acne. However, with the understanding that the brain–gut–skin axis exists, it is now clear that intestinal microbes have significant effects on acne. As understanding of the microbiome in healthy skin and the pathophysiology of acne continues to develop, new therapeutic targets are arising. Novel systemic and topical interventions that influence the microbiota (i.e., probiotics, prebiotics), custom tailored to each patient according to their unique microbial ‘fingerprint’, are worthy of intense research.

## Figures and Tables

**Table 1:** Comparison of the different skin sampling methods.

	Sampling Method			
	Swab	Scrape	Pore Strip	Biopsy
*C. acnes* populations				
Superficial stratum corneum	+	+	+	± ^a^
Within stratum corneum	–	+	+	+
Infundibulum	–	–	+	+
Lower hair follicle	–	–	–	+
Follicular biofilms	–	–	–	± ^b^
Advantages and disadvantages				
Pros	Simple, quick, and noninvasive.	Enables collection of skin cells and their associated microbes.	Collects follicular contents.	Samples all layers of the skin.
Cons	Might not correctly reflect the microbiota across all skin layers.	Might not correctly reflect the microbiota across all skin layers.	Might not reflect the microbiota in the lower hair follicles.	Is invasive and covers a smaller surface area than the other sampling methods.

^a^ Likely removed during preparation of the field with antiseptics; ^b^ Likely requires special pre-treatment, e.g., sonication prior to DNA extraction and sequencing.

**Table 2:** Summary of the nomenclatures of *C. acnes* phylotypes and their association with acne and healthy skin.

Clade (Based on Whole-Genome Sequencing)	Clade (Based on Belfast eMLST [38])	Clade (Based on Aarhus MLST [39])	RT [30]	Acne	Healthy Skin
IA-1	IA1	I-1a	RT1	√	√
IA-2	IA1	I-1a	RT4, RT5	√	
IB-1	IA1	I-1b	RT8	√	
IB-2	IA2	I-1a	RT3	√	√
IB-3	IB	I-2	RT1	√	√
IC	IC	NA	RT5	√	
II	II	II	RT2, RT6		√
III	III	III	NA		

eMLST: expanded multi-locus sequence typing; MLST: multi-locus sequence typing; NA: not assigned; RT ribotype.

**Table 3:** Skin and gut microbiota of acne patients compared with healthy controls.

Significant Changes in Skin Microbiota	Significant Changes in Gut Microbiota
↑*Cutibacterium acnes* ↑*Cutibacterium granulosum* ↑*Staphylococcus epidermidis* ↑*Proteobacteria and Firmicutes* ↓*Actinobacteria* ↑*Sterptococcus* (pre-adolescent) ↑*Malassezia species*	↑*Bacteroides*

**Table 4:** Probiotics and acne.

Key Microbes Involved	Potentially Beneficial Microorganisms	Main Mechanism of Action	Experimental Model
*C. acne* (hyper-colonization and dominance of virulent strains)	*Staphylococcus epidermidis* [18]	Fermentation of glycerol (inhibition of *C. acnes* growth)	In vitro
	*Streptococcus salivarius* [166]	Production of bacteriocin-like inhibitory substance (inhibition of *C. acnes* growth)	In vitro
	*Lactococcus sp. HY449* [167]	Release of bacteriocin (inhibition of *C. acnes* growth)	In vitro
	*Streptococcus thermophiles* [169,170]	Increase in ceramide production, secondary antimicrobial activity (restoration of the skin barrier, inhibition of *C. acnes* growth)	In vivo, In vitro
	*Lactobacillus paracasei* [173,174]	Suppression of substance P-induced inflammation (reduction of inflammation)	Ex vivo
	*Enterococcus faecalis* [178]	Production of enterocins (inhibition of *C. acnes* growth)	In vivo
	*Lactobacillus plantarum* [179]	Production of antimicrobial peptides (inhibition of *C. acnes* growth)	In vivo
